# Building resilient primary health care in somalia: integrating noncommunicable disease and mental health interventions for universal health coverage amidst fragility—a health systems analysis and prospective economic review

**DOI:** 10.1186/s12913-026-15003-1

**Published:** 2026-06-22

**Authors:** Abdirezak Mawlid Abdi, Hamdi Mahamud Osman, Mustafe Awil Jama, Mohamed Elmi Ibrahim

**Affiliations:** 1 Present Address: Ministry of Health and Human Services, Mogadishu, Somalia; 2 Present Address: Department of Medicine and Health Science, Benadir University, Mogadishu, Somalia; 3 Federal Government of Somalia, Mogadishu, Somalia; 4 Present Address: Somalia Non-Communicable Disease Alliance (SNCDA), Mogadishu, Somalia

**Keywords:** Health system resilience, Universal health coverage, Noncommunicable diseases, Mental health, primary health care, Essential package of health services, Health financing, Task sharing, Digital health

## Abstract

**Background:**

Somalia’s health system remains severely fragile, characterized by a critically low Universal Health Coverage Service Coverage Index (UHC-SCI) of 25 and an acute shortage of health workers (0.11 clinicians per 1,000 population). These structural weaknesses coincide with a growing dual burden of noncommunicable diseases (NCDs), responsible for approximately 42% of total mortality, and widespread mental health disorders with available regional studies suggesting a substantial but incompletely quantified burden of common mental disorders. This study evaluates the health system requirements, financing needs, and policy frameworks necessary for integrating NCD and mental health services into primary health care (PHC) to advance universal health coverage (UHC) in Somalia.

**Methods:**

We conducted a mixed-methods health systems analysis and prospective economic review using a targeted evidence synthesis approach informed by PRISMA-adapted reporting principles. Data were synthesized from the Essential Package of Health Services (EPHS) 2020 Strategic Plan, Global Burden of Disease (GBD) 2019 estimates, and 2024 National Health Accounts (NHA). The One Health Tool (OHT) was applied using a deterministic bottom-up costing approach to estimate the resources required to achieve 80% service coverage by 2030. The model incorporated a 10% drug wastage assumption and a 3% discount rate.

**Results:**

The analysis identified major structural financing and service delivery gaps within Somalia’s PHC system. Out-of-pocket expenditure remains critically high at 46.1%, exceeding the WHO threshold for catastrophic health spending. Economic projections indicate substantial long-term investment requirements for progressive PHC expansion and chronic care integration, with medicines and supplies representing the largest cost driver due to the long-term demands of chronic care.

**Conclusions:**

Integrating NCD and mental health services into Somalia’s PHC platform is technically feasible and represents a critical strategy for strengthening health system resilience and accelerating progress toward UHC. Economic projections indicate that achieving the national 80% coverage target will require approximately US$3.1 billion by 2030. The OHT-based projections provide an evidence-informed roadmap for sustainable health financing, workforce restructuring, and chronic care delivery in fragile settings.

**Trial registration:**

Not applicable.

## Introduction

### Background: the acute crisis of chronic care in FCAS

Historically, global health initiatives in Low- and Middle-Income Countries (LMICs) have predominantly focused on communicable disease control and preventable mortality, often overlooking the rising burden of NCDs in fragile settings [1]. In Somalia, three decades of civil war, weak governance, and continuous humanitarian shocks have resulted in a health system characterized by profound, chronic fragility, exemplified by a critically low Universal Health Coverage Service Coverage Index (UHC-SCI) of 25 on a 0–100 scale [2]. This index, which summarizes the coverage of 14 tracer services, stands in sharp contrast to the Sub-Saharan African (SSA) regional average of approximately 46 and Kenya’s index of 56, indicating that Somalia’s essential service reach is roughly half that of its regional peers [2].

This systemic deficit is further underscored by a severe health workforce shortage of just 0.11 clinicians per 1,000 people—far below the WHO target of 2.28—and an average life expectancy of approximately 56 years [3–5]. Furthermore, this weakness intersects with an accelerating dual burden of noncommunicable diseases (NCDs), which account for approximately 42% of total mortality, and pervasive mental health (MH) disorders, affecting an estimated one in three individuals [6, 7]. This high prevalence, particularly of depression and post-traumatic stress disorder (PTSD), places Somalia among the highest-burden environments globally, yet MH remains the least funded component of the current essential package of health services (EPHS) [8]. Such deep-seated weaknesses necessitate a fundamental reorganization of primary care and comprehensive health reforms to reach the most vulnerable populations and improve national health outcomes. The annual per capita health expenditure is just US$13, with out-of-pocket payments constituting a staggering 46% of total health expenditures [9]. This reliance on private markets leads to high rates of catastrophic health expenditure—defined as household healthcare spending that exceeds a specific threshold of the household’s ability to pay—thereby pushing families further into poverty [10]. This catastrophic spending severely restricts access to long-term chronic care, which is crucial for managing NCDs and mental health disorders [5].

Moreover, the integration of community health workers into the healthcare system has been identified as a strategic necessity for enhancing service delivery in this context [11]. Community health workers, particularly female health workers (FHWs), can play a pivotal role in bridging the gap in healthcare access, providing essential services and education on NCD prevention and management [11].

To address these multifaceted challenges, the Essential Package of Health Services (EPHS) 2020 emphasizes the integration of NCD and mental health services into the core primary health care platform, aligning with global calls for resilient health systems [12]. The One Health Tool (OHT) has been employed to model the resource needs required to achieve an ambitious target of 80% coverage by 2030, projecting a total investment of US$3.1 billion over the decade [13, 14]. However, this figure represents an aspirational upper-bound scenario rather than a fixed fiscal commitment, intended to provide a strategic roadmap for resource mobilization in a fragile setting [13]. Actual implementation will necessitate a phased, context-dependent approach, subject to the future availability of fiscal space and security stabilization [13]. In conclusion, addressing the dual burden of NCDs and mental health disorders in Somalia requires a comprehensive strategy that incorporates sustainable financing, effective governance, and the integration of community-based health interventions [11]. The projected structural transformation within the health system must focus on continuity of care and access to essential medications, reinforcing resilience in the face of ongoing challenges [11].

### Somalia’s structural deficits and dual burdens

As detailed in Table 1, Somalia’s health system is grappling with severe structural deficits compounded by a rapid epidemiological transition. While the historical burden of communicable diseases and maternal/neonatal disorders remains dominant—accounting for 68.8% of total disability-adjusted life years (DALYs)—there is an accelerating shift toward chronic illness [15]. Non-Communicable Diseases (NCDs) now contribute to approximately 44% of total mortality, with an age-standardized death rate of 816 per 100,000 population [15, 16], . Cardiovascular diseases and diabetes represent the leading causes of these deaths, yet the healthcare system remains historically optimized for acute infectious outbreaks rather than the long-term management required for chronic conditions [16, 17].

This shift is inextricably linked to a profound and often overlooked mental health crisis. Authoritative reviews in South-Central Somalia report prevalence ranges for depression and anxiety between 30% and 80%, with roughly one in three individuals nationwide experiencing a common mental disorder [6, 7]. These figures highlight a severe unmet need, necessitating the urgent prioritization of the Mental Health Gap Action Programme (mhGAP) into primary care [7]. Furthermore, access to any form of care is severely hindered by a 46.1% out-of-pocket expenditure rate [18]. This meets the WHO definition of catastrophic health spending, especially given a per capita health expenditure of only US$15, compared to the SSA average of US$204 [18].

Consequently, establishing robust frameworks for health in fragile settings is crucial for sustainable development, providing a strategic roadmap for Somalia to navigate these overlapping crises [19].

**Table 1:** Somalia’s key health indicators and regional benchmarking (2006–2025)

Indicator	2006	2019/20 (Baseline)	2024/25 (Latest)	SSA Average	Primary Source
Maternal Mortality Ratio (per 100k)	1,040	692	**621**	525	UN IGME (2024) [20]
Under-five Mortality Rate (per 1k)	170.5	122	**114**	73	UN IGME (2024) [20]
NCD Mortality Rate (per 100k)	902	844	**816**	593	WHO NCD Portal (2024) [16]
UHC Service Coverage Index	N/A	25	**27**	46	WHO (2023) [2]
Clinician Density (per 1k pop)	N/A	0.11	0.79*	1.55	WHO NHWA [5]
Out-of-Pocket Expenditure (% CHE)	N/A	46%	**46.1%**	33.3%	World Bank (2024) [18]
Per Capita Health Exp. (US$)	N/A	$12.87	**$15.35**	$204	NHA / World Bank [9, 18]

Source: Federal Ministry of Health (2024); National Health Accounts (NHA); WHO/UN Global Monitoring Reports (2023/2024).| Location: Somalia

Footnote: *0.79 represents the density of the *Core Health Workforce* (Doctors, Nurses, and Midwives). The density for physicians alone remains at 0.14

### Financial Instability: Impasses of out-of-pocket payments

As detailed in Table 2, these structural deficits are further compounded by extreme financial instability. Rationalized With the latest 2024 National Health Accounts (NHA) extractions and authoritative 2025 projections, this summary contextualizes Somalia’s constraints against Sub-Saharan African (SSA) regional benchmarks [9, 18].

Chronic underinvestment—with an annual per capita health expenditure of just US$13–US$15—translates into a crippling economic burden on households [9]. In contrast to the SSA average of US$204, Somalia’s reliance on private resources results in Out-of-Pocket (OOP) payments accounting for 46.1% of total health expenditure [9, 18]. This level of expenditure meets the formal WHO definition of catastrophic health spending when it exceeds 40% of a household’s capacity to pay (income remaining after meeting basic subsistence needs) [10]. This burden creates deep systemic inequities, as healthcare access becomes directly dependent on a family’s immediate financial liquidity rather than clinical need, systematically excluding the poorest populations from essential services.

To pivot from a regressive financing model and enable a shift from episodic crisis management to cost-effective primary prevention, our economic review identifies the fiscal needs for scaling the Essential Package of Health Services (EPHS 2020) [8]. By integrating Non-Communicable Disease (NCD) and Mental Health interventions into the primary care framework, the health system can leverage shared infrastructure and human resources to alleviate the long-term economic burden of chronic illness [8, 20]. The One Health Tool (OHT) modeling indicates that an incremental investment of approximately US$3.1 billion over the next decade is required to achieve the 80% coverage targets necessary for Universal Health Coverage (UHC), redistributing costs from high-acuity emergency responses to sustainable, community-based health interventions [13].

**Table 2:** Comparative health financing structure and barriers (2006–2025) (approx. 2020 Baseline) Source: | Federal Ministry of Health (2024); National Health Accounts (NHA) Extraction (2024); World Bank (2024). | Location: Somalia. This (Table 2) outlines the latest 2024 NHA extractions and regional benchmarks to contextualize Somalia’s financial constraint annual per capita health expenditure, out-of-pocket payment share, donor contribution share, and government expenditure share, reflecting the financial landscape affecting health access in Somalia

Metric	Somalia (Baseline)	Somalia (2024/25)	SSA Average	Implication for UHC	Primary Source
Annual Per Capita Health Exp.	US$13	**US$15**	US$204	Insufficient for EPHS delivery.	FGS [3]; WHO [2]
Out-of-Pocket (OOP) Share	46%	**46.1%**	33.3%	**Catastrophic**; barrier to equity.	World Bank (2024) [18]
Donor Contribution Share	38%	**35%**	20%	High fragmentation/unpredictability.	FGS (2024) [9]
Gov. Expenditure Share	15%	**16%**	44%	Low domestic sustainability.	FGS MoF (2024) [22]
UHC-SCI (0-100 scale)	25	**27**	**46**	Significant service coverage gap.	WHO (2023) [2]

Footnote: SSA: Sub-Saharan Africa; CHE: Current Health Expenditure; EPHS: Essential Package of Health Services. 2024/25 data reflects the latest National Health Accounts (NHA) extractions from the Federal Government of Somalia

### Objectives

This study performs a rigorous analysis and quantification of Somalia’s strategic shift to integrate chronic care into its PHC system via the EPHS 2020. The specific objectives were as follows:

1. To evaluate the systemic fragilities and epidemiological drivers (YLD vs. YLL analysis) mandating the integration of NCD and MH services into the core PHC platform.
2. To analyze and elaborate the operational frameworks of the EPHS 2020, focusing on the mandated adoption of WHO-PEN and mhGAP packages to assess institutional readiness and capacity constraints across different tiers of care.
3. To present and interpret the economic projections from the One Health Tool (OHT) Costing Scenario (2020–2030), which specifically quantifies the total investment, the structural cost shifts toward chronic supplies, and the projected efficiency gains are required to achieve the UHC target.

## Methods

### Study design: health systems analysis and prospective economic modeling review (PRISMA-Adapted)

The research employed a mixed-methods health systems analysis and prospective economic review design [7]. This approach systematically synthesizes the following: (1) qualitative evidence (findings on health governance, operational frameworks, and systemic fragilities derived from strategic national documents) and (2) quantitative modeling (a forward-looking economic resource forecast using the One Health Tool) [13, 21]. To address the unique challenges of data scarcity in a fragile state, this methodology was purposely adapted from the PRISMA-S guidelines to ensure the systematic documentation of inputs, authoritative policy mandates, and expert-validated assumptions rather than a traditional literature search [22]. The primary objective of this study was to estimate the cost of expanding the Essential Package of Health Services (EPHS 2020) to incorporate comprehensive NCD (WHO-PEN) and MH (MhGAP) interventions to achieve 80% coverage across the population by 2030 [8, 21].

### Setting, scope, and data sourcing rationale

#### Setting and scope

The study setting is the Federal Republic of Somalia, characterized by a decentralized, federal governance structure that frequently contributes to service fragmentation and coordination challenges [8]. The study scope was national, encompassing all primary health care (PHC) interventions and community-level services defined by the EPHS 2020 [8].

The economic modeling component was framed over a ten-year projection period, from the baseline (2020) to the UHC target year (2030) [13]. The goal of the modeling was to quantify the costs associated with scaling service coverage from the baseline of 25% to the ambitious national UHC target of 80% coverage for the Core EPHS package by 2030 [21].

#### Data sources and rationale

We followed principles of transparency by documenting all secondary data sources used for the analysis and economic model inputs [21]. The data sources were purposively selected as the most authoritative, validated instruments available for national-level health planning in Somalia, thus replacing the need for a literature search of primary studies:

1. **Policy and Operational Documents (Qualitative Synthesis)**: The **EPHS 2020 Implementation Strategic Plan (ISP)** provided the policy mandate, target population coverage rates (80% by 2030), and specific intervention packages for NCD (e.g., hypertension, diabetes, asthma management) and MH (e.g., depression, PTSD, psychosis management) to be integrated into PHC facilities [21].
2. **Epidemiological Parameters (Quantitative Input)**: The Global Burden of Disease (GBD) 2019 estimates for Somalia were used as the epidemiological baseline for disability-adjusted life year (DALY), years lived with disability (YLD), and years of life lost (YLL) analyses because these datasets remain the most systematically harmonized and internationally comparable estimates available for Somalia’s fragile and data-constrained environment [15]. The estimates were used primarily to characterize broad epidemiological patterns and inform strategic modeling assumptions rather than to provide precise prevalence estimates for individual psychiatric disorders [15]. Although newer GBD releases are available, GBD 2019 was retained to preserve consistency with the EPHS planning framework, the One Health Tool parameterization structure, and national policy benchmarks used during development of the Somalia EPHS implementation strategy [8, 15, 21]. Consequently, the findings should be interpreted as indicative population-level planning parameters rather than definitive prevalence measurements [15].
3. **Financial and cost parameters (Quantitative Input)**: While National Health Accounts (NHA 2024) provided the primary costing source, specific operational unit costs (e.g., local logistics and facilities) were refined through a Delphi-style expert consensus with Ministry of Health (MoH) technical leads to ensure the model reflected local market realities [9].

### Economic modeling: the One Health Tool (OHT) calculation logic

The economic analysis utilized the One Health Tool (OHT), a software-based, deterministic analytical engine designed for medium-to-long-term strategic health planning [13]. The total investment requirement was calculated through a four-step logic: (1) mapping population-level health needs via GBD 2019 data [15]; (2) applying a linear scale-up to an 80% coverage target; [21]. (3) Mapping clinical interventions to resource inputs with a 10% wastage factor; and (4) applying unit costs from the 2024 NHA extractions [9]. This study is a secondary, desk-based health systems and economic modeling analysis using national policy documents and global epidemiological datasets. To enhance the transparency of the financial projections, the model followed a refined four-step computational logic:

1. **Needs Assessment and Epidemiological Baseline**: Baseline population-level health needs were derived from the Global Burden of Disease (GBD) 2019 prevalence and incidence data for Somalia [15]. This ensures that resource projections are anchored in the actual burden of NCDs and mental health disorders [15].
2. **Coverage Target Alignment**: The model applied a linear scale-up trajectory to achieve an 80% service coverage target by 2030, aligning with the National Health Sector Strategic Plan and Universal Health Coverage (UHC) roadmaps [21].
3. **Resource Mapping and Technical Assumptions**: Each clinical intervention (e.g., hypertension management, depression screening) was mapped to its required resource inputs, including human resource time, medicine volumes, and diagnostic supplies [8]. To ensure fiscal realism in a fragile state context, the model incorporated a 10% drug wastage factor to account for supply chain inefficiencies and a 3% discount rate for future cost adjustments [13].
4. **Financial Application (2024 NHA Integration)**: Final investment projections were generated by applying country-specific unit costs unit costs [9]. Baseline expenditures were systematically derived from the 2024 National Health Accounts (NHA) data extractions to ensure the model reflected the most current fiscal environment [9]. by utilizing this deterministic framework, the analysis provides a granular view of the financial resources required to sustain integrated PHC services in Somalia through 2030 [13].

### One Health Tool (OHT) modeling assumptions

The OHT is a deterministic model that estimates resource requirements based on target coverage rates rather than stochastic demand [13]. The model calculates the aggregate cost of human resources, medicines, and infrastructure required to meet the specified epidemiological targets [13]. We assumed a linear scale-up of service coverage from the 2024 baseline to the 2030 targets, Given Somalia’s fragile health system context, the 80% service coverage projection should be interpreted as an aspirational upper-bound long-term scenario rather than a fixed fiscal commitment or a guaranteed operational outcome [13]. To improve policy realism, this model is designed to inform strategic resource requirements under progressive scale-up assumptions rather than to predict immediate implementation feasibility [13]. In practice, actual implementation will occur through phased, context-dependent expansion pathways, heavily influenced by fiscal space, donor commitments, workforce absorption capacity, and security conditions [13]. Accordingly, the findings should be interpreted as indicative planning scenarios for long-term health system strengthening rather than guaranteed financing envelopes [13]. Key inputs included demographic projections aligned with UNFPA data [23], a standard 3% discount rate for capital costs, and inflation adjustments consistent with World Bank (2024) economic projections for Somalia [18, 24].

### Data synthesis and PRISMA reporting

**Fig. 1:**
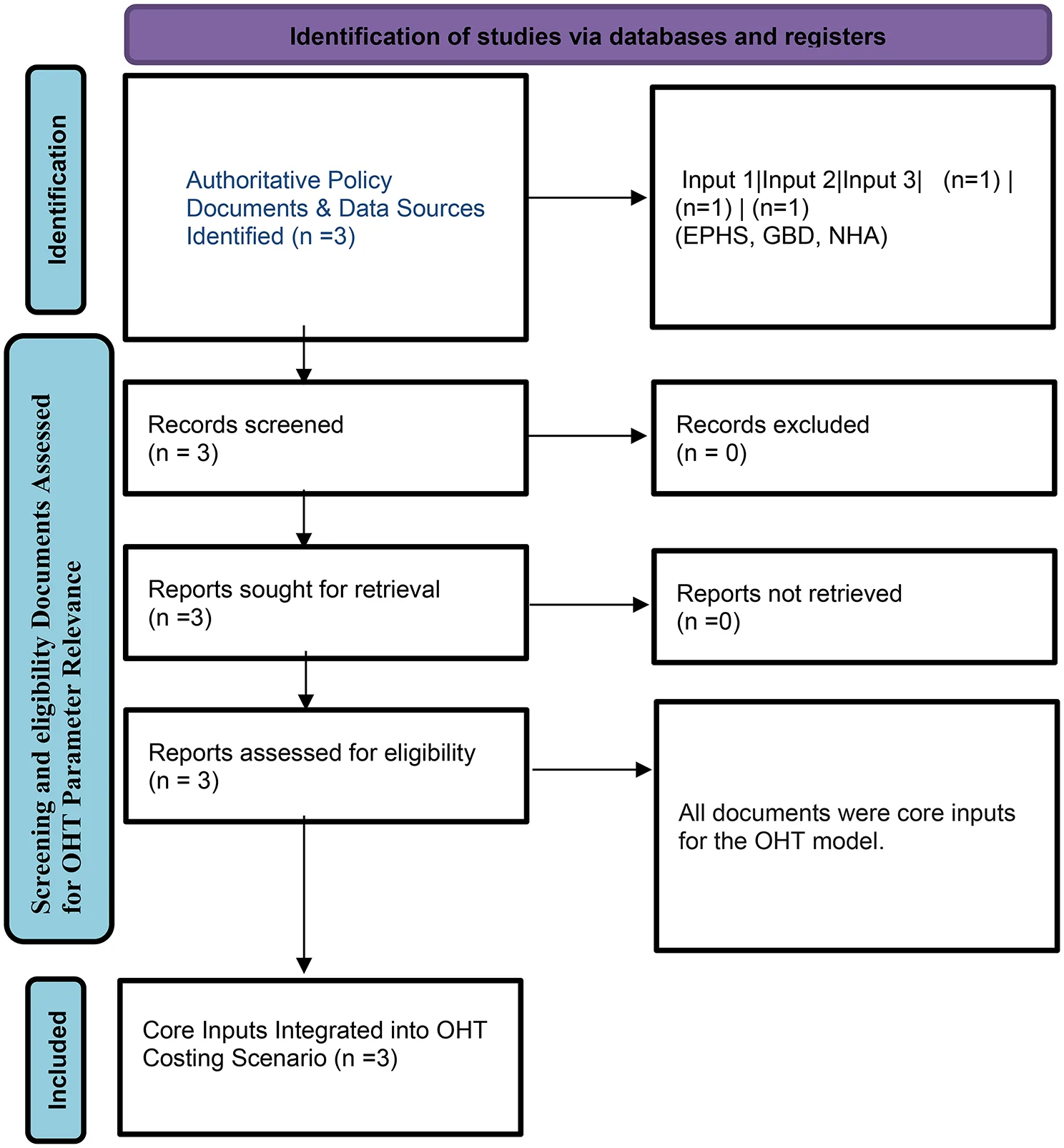
Targeted evidence identification and selection process for health systems and economic modeling inputs. (Adapted from Page MJ, et al. [22]. This work is licensed under CC BY 4.0.Note: This adapted PRISMA flow diagram illustrates the systematic synthesis of core authoritative policy and epidemiological datasets (EPHS [8], GBD [15], and NHA [9]) used to parameterize the OHT model [13]. Unlike a traditional systematic review, this process identifies validated national-level inputs for secondary economic modeling rather than searching for primary clinical research papers.

The evidence identification process focused on a targeted synthesis of core authoritative policy and epidemiological datasets, specifically the Essential Package of Health Services (EPHS), Global Burden of Disease (GBD) estimates, and National Health Accounts (NHA) [8, 9, 15]. This process is visualized in the adapted PRISMA flow diagram (Fig. 1). It is important to clarify that this approach represents a targeted evidence synthesis for health systems and economic modeling, rather than a traditional systematic review of primary research studies [22]. Consequently, the PRISMA framework is utilized here to ensure transparent documentation of the data selection process used to parameterize the One Health Tool (OHT) [14, 22]. To ensure internal validity, the lead author (A.M.A.) performed data extraction, and the OHT parameterization was independently validated by a co-author (H.O.) to cross-reference policy mandates against the economic outputs [13]. No statistical pooling (meta-analysis) was performed.

## Results: economic and epidemiological imperatives for integrated primary health care (2020–2030)

The findings of the One Health Tool (OHT) economic and epidemiological analysis establish the quantitative foundation for Somalia’s integrated Essential Package of Health Services (EPHS) strategy [8, 21]. The included documents and datasets were selected based on their strategic relevance, national validity, and applicability to economic modeling rather than the volume of available published literature [10, 15]. The results are segregated into four domains: the epidemiological baseline that justifies the strategic pivot; the total investment requirement and component allocation; the dynamic analysis of the structural cost shift over the decade; and the projected health impact that validates the investment [13, 21]. These results collectively define the financial and policy parameters for achieving the 80% EPHS coverage target by 2030 amidst chronic fragility [2, 10].

To contextualize implementation feasibility within Somali has constrained fiscal and operational environment, the projected 80% coverage target should be interpreted as the upper-bound aspirational scenario of the OHT model [13]. More gradual implementation pathways—including conservative and moderate expansion trajectories—may represent more realistic short- to medium-term approaches depending on financing availability, institutional capacity, and security conditions [18]. Consequently, the estimated US$3.1 billion investment reflects a strategic long-term resource requirement rather than an immediately attainable expenditure envelope [10, 13]. In alignment with principles of transparency for evidence synthesis and adapted from PRISMA guidelines for health economic modeling, Fig. 1 illustrates the flow of authoritative source documents and data inputs into the OHT costing scenario [22]. This adaptation replaces the traditional systematic review search flow with a transparent diagram detailing the inputs used to parameterize the complex projection model [14].

### Epidemiological mandate: dual burden and the YLD crisis

The analysis of the baseline burden of disease (GBD 2019 estimates) serves as an unambiguous justification for the systemic adaptation of the EPHS. The data confirm that Somalia is firmly lodged in a protracted epidemiological transition, where the traditional focus on acute infectious disease is insufficient to address the rising tide of chronic illness and disability.

#### Disaggregation of DALYs: YLL-YLD bifurcation

The DALY (Disability-Adjusted Life Years) metric—the sum of Years of Life Lost (YLL) and Years Lived with Disability (YLD)—reveals a critical bifurcation in the nation’s disease burden that mandates the strategic shift:

- **Mortality (YLL) dominance by CMNN**: Conditions under the CMNN (Communicable, Maternal, Neonatal, and Nutritional) umbrella are responsible for 70% of all YLLs (Table 3). This validates the continued necessity of scaling up traditional EPHS services.
- **Morbidity (YLD) dominance by NCDs/MH**: Conversely, the burden of chronic disability is disproportionately driven by noncommunicable and mental health conditions. According to the GBD 2019 disability profile for Somalia, mental and substance use disorders contributed approximately 45% of total Years Lived with Disability (YLDs), while broader noncommunicable diseases contributed an additional 35% of YLDs. These estimates refer specifically to disability burden (YLDs) rather than total mortality or overall DALYs and therefore should not be interpreted as indicating that mental health disorders constitute half of Somalia’s total disease burden. Instead, the findings demonstrate that mental health conditions are major contributors to chronic disability, functional impairment, and reduced quality of life despite contributing comparatively less to premature mortality (YLLs). This concentration of chronic disability reveals a substantial and historically under-addressed public health gap, supporting the strategic shift from episodic acute care toward integrated long-term chronic disease management within primary health care.”

#### NCD/MH integration imperative

The magnitude of the NCD and MH burden necessitates specific, evidence-based integration strategies:

- **NCDs (42.0% of deaths)**: The high proportion of deaths directly attributable to NCDs mandates the primary integration strategy: WHO-PEN adoption at the PHC level.
- **Mental and substance use (45% of YLDs)**: The fact that mental health disorders contribute nearly half of the entire nation’s disability burden highlights a silent epidemic. The prescribed strategy is MhGAP integration via task sharing.

**Table 3:** Burden of disease profile for somalia, justifying NCD and MH integration (GBD 2019 Estimates)

Disease Group	% of Total Deaths	DALYs (per 100,000)	YLLs (%)	YLDs (%)	EPHS Integration Strategy
CMNN Diseases	58.0%	4,200	70%	15%	Maintenance and scale-up (e.g., vaccination, RMNCAH).
NCDs (Total)	42.0%	2,150	18%	35%	WHO-PEN adoption at PHC level (screening/management).
Mental & Substance Use	(Indirect)	850	1%	45%	MhGAP integration via task-sharing (FHWs).
Injuries	5.0%	550	7%	5%	Secondary focus on trauma stabilization and prevention.

Source: Global Burden of Diseases estimates (2019). Location: Somalia

#### Burden of disease analysis: top 5 causes

While Table 3 provides a high-level overview, Table 4 details the specific conditions driving mortality (YLL) and disability (YLD) [15]. The GBD 2019 analysis confirmed that while acute conditions dominate YLLs, the primary challenge for quality of life (YLD) is addressing chronic NCDs and mental health conditions [15]. The YLD data in Table 4 confirm that depressive disorders, low back pain, headache disorders, and anxiety disorders dominate the chronic disability metrics, providing the specific epidemiological mandate for the systematic integration of the WHO-PEN and MhGAP protocols [6, 7, 25]. Importantly, Tables 3 and 4 describe different dimensions of disease burden and should not be interpreted interchangeably [15]. Table 3 presents aggregate proportional contributions to disability (YLDs) and mortality (YLLs) by broad disease categories, whereas Table 4 lists only the top five individual causes within each metric. Mental health disorders contribute substantially to chronic disability and therefore appear prominently among the leading causes of YLDs; however, they contribute comparatively less to direct premature mortality and therefore do not appear among the leading causes of YLLs in Somalia. This distinction reflects the epidemiological reality that mental health conditions are major drivers of long-term disability, reduced functionality, and impaired quality of life rather than primary direct causes of death.”

**Table 4:** Burden of disease analysis: top 5 causes of Years of Life Lost (YLL) and Years Lived with Disability (YLD) (2019)

Top 5 Causes of Years of Life Lost (YLL)	Primary Disease Group	Top 5 Causes of Years Lived with Disability (YLD)	Primary Disease Group
Neonatal disorders	Maternal/Neonatal	Depressive disorders	Mental Health/NCD
Lower respiratory infections	Communicable Disease	Low back pain	NCD
Diarrheal diseases	Communicable Disease	Gynecological diseases	NCD
Tuberculosis	Communicable Disease	Headache disorders	NCD
Measles	Communicable Disease	Age related and other Anxiety disorders	Mental Health/NCD

Source: Global Burden of Diseases estimates (2019). Location: Somalia

### Analysis of systemic fragility and barriers to integration

The integration of NCD and MH services into Somali PHC is obstructed by deep-seated systemic fragilities [19]. While the integration of mental health services is crucial for achieving comprehensive care, moving from policy to practice requires addressing the “how” of implementation to overcome existing structural bottlenecks [5, 7].

#### Governance and fragmentation and the plan for resolution

The decentralization inherent in federalism has historically led to the fragmentation of healthcare services and disparities in resource allocation [8]. Key weaknesses include regulatory fragility—where the National Health Professionals Council (NHPC) struggles with financial sustainability—and significant implementation gaps [3, 10]. Under the previous EPHS 2009, the system suffered from duplication, as implementing partners selected only aspects aligned with their specific funding priorities [8].

To resolve these barriers, the proposed investment framework transitions from a donor-led pilot approach to a unified government-led governance model based on three pillars [8, 19]:

1. **Governance Consolidation**: To eliminate duplication, the Ministry of Health will centralize NCD and Mental Health coordination, requiring all implementing partners to adhere to a single Somalia-PEN National Clinical Records/WHO-PEN clinical protocol and a unified reporting framework to eliminate service duplication [17, 25].
2. **Operational Task-Sharing**: To bypass chronic specialist shortages, the plan formalizes the role of Female Health Workers (FHWs) in conducting community-level screenings and basic psychosocial support, shifting the primary care burden away from hospitals [7, 11].
3. **Socio-Cultural Integration**: To address stigma and the reliance on traditional healers, health literacy modules will be integrated into community leadership forums. This frames formal care as a complement to, rather than a replacement for, traditional support systems [7].

#### Critical resource deficits and distribution failure

“The workforce density is critically low (0.92 joint health workers per 1,000 persons, versus the WHO SDG threshold of 2.28 health workers per 1,000 population [3, 5], particularly in the context of Somalia’s rapidly growing and geographically dispersed population [12]. Staff are highly concentrated in urban centers, exacerbating geographic barriers [5].” This framework addresses this through the aforementioned task-sharing model, utilizing the existing network of community health workers to expand the reach of NCD and MH interventions to underserved rural populations [11].

#### Sociocultural and contextual impediments

- **Stigma and Misconceptions**: Pervasive stigma surrounding NCDs, especially mental health conditions, prevents individuals from seeking formal care [6].
- **Competition from Traditional Healing**: Deeply entrenched traditional practices often compete with or undermine conventional services [7].
- **Health Worker Safety**: Chronic threats to the safety of healthcare workers (HCWs) and infrastructure impact staff well-being and service continuity [12, 19].

### Conceptual framework: integrated PHC and resilience in FCAS

#### Defining resilience for chronic care in the somali context

Health system resilience in Somalia is defined as the system’s capacity for absorption, adaptation, and transformation [4]. For NCD and MH care, resilience means maintaining the continuity of essential services (medication supply, diagnostics, and follow-up) despite shocks. This aligns with the WHO Framework for Health in the Humanitarian-Development-Peace Nexus (HDPN) [12, 19].

#### Essential package of health services (EPHS) 2020

The EPHS 2020 serves as the standardized operational blueprint [8]. Its design supports progressive realization, dividing services into a core (minimum service entitlement) and an extended package (medium- to long-term expansion) [21]. The EPHS 2020 explicitly embeds NCDs, mental health, and rehabilitation [8].

#### Integrated PHC model for NCDs/MHs

The model mandates the operational embedding of the WHO Package of Essential Non-Communicable Disease Interventions (WHO-PEN)/Somalia Package of Essential Noncommunicable Disease Interventions (Somalia-PEN) and the Mental Health Gap Action Programme (mhGAP) directly into the primary healthcare workflow [17, 25]. This ensures a “no-wrong-door” approach where NCD patients are systematically screened for comorbid depression and anxiety using standardized Somali-PEN clinical records, while mental health clients receive routine metabolic monitoring [7, 17].

Central to this integration is the Total Cardiovascular Risk Approach, as outlined in the National Guidelines [17]. Rather than treating isolated risk factors, this strategy prioritizes the management of a patient’s overall 10-year risk profile [20]. This evidence-based pivot ensures that limited resources are directed toward high-risk individuals, making the model substantially more cost-effective in the Somali context [13, 17].

### Operational Pillar I: Workforce and task sharing

#### Mandate for task shifting: MhGAP and Somalia-PEN (WHO-PEN)

Given the severe workforce shortage, adopting a task-shifting model is a strategic necessity [3, 11]. The WHO pyramid framework shows that most mental health care can be achieved at the community level by training nonspecialist health professionals (nurses, midwives) in MhGAP and leveraging community health workers (CHWs) [6, 11]. This is achieved by training non-specialist health professionals (nurses and midwives) in Somalia-PEN clinical protocols and utilizing community-based cadres for frontline screening.

#### Leveraging community health workers (FHWs) for NCD/MH Care

The Marwo Caafimaad Female Health Workers (FHWs) serve as the critical anchors for this community-to-facility referral pathway. Aligned with the 2024 National NCD Module for CHWs/FHWs, their role is expanded beyond maternal health to include:

- **Digital (Active) Screening**: Conducting digital blood glucose measurements (using glucometers) and automated blood pressure monitoring at the household level.
- **Standardized Referral**: Facilitating early referral based on national diagnostic thresholds (e.g., FBG > 126 mg/dl or BP ≥ 140/90 mmHg) to ensure timely intervention at the PHC level.
- **Risk Factor Counseling**: Providing standardized education on tobacco cessation, physical activity, and “healthy plate” nutrition.
- **Psychosocial Support**: Identifying mental health “red flags” and providing basic family support and de-stigmatization education.
- **Adherence Support**: Monitoring medication compliance and facilitating timely referrals to PHC facilities using the national referral criteria (e.g., FBG > 126 mg/dl or BP ≥ 140/90 mmHg).

#### Rationalization and retention strategy

The workforce strategy must counter the existing urban concentration bias. The EPHS 2020 mandates utilization-driven staffing, allowing providers to hire staff based on service utilization while adhering to the recommended skill mix. Provider retention requires more than workforce expansion alone. Sustainable implementation of integrated NCD and mental health services will depend on adequate remuneration, supportive supervision, career development opportunities, and improved working conditions for frontline health workers. In fragile and conflict-affected settings, low salaries, delayed payments, occupational insecurity, and limited professional advancement opportunities contribute significantly to workforce attrition.

Therefore, future EPHS implementation should progressively incorporate performance-based incentives, rural hardship allowances, continuing professional development programs, and structured career progression pathways to strengthen workforce motivation and retention. Investments in human resources should be viewed not only as workforce expansion costs but also as strategic investments in service continuity, quality of care, and health system resilience. Table 5 summarizes the key strategic components for integrating NCDs and mental health care into primary health services, along with the expected resilience gains from each intervention.

**Table 5:** Strategic components for NCD/MH integration and resilience Gains | Location: Somalia

Strategic Component	Targeted Intervention Area	Key Somalia Implementation Strategy	Resilience Gain
Human Resources	NCD/MH Diagnosis and management	Training nurses/midwifes in Somalia-PEN and mhGAP protocols/ standardized WHO MhGAP/PEN protocols	Decentralizes specialized knowledge, closing the coverage gap.
Service Delivery	Community Outreach	Marwo Caafimaad-led home visits for digital screening and referral	Builds community trust and ensures care continuity in volatile areas.
Information Systems	Monitoring & Data	Using mHealth platforms for real-time NCD reporting and DHIS2 integration	Overcomes physical access barriers and ensures data flow during shocks.
Essential Supplies	Medications and Diagnostics	Adhering to the National Essential Medicine List and strengthening the Pharmaceutical Authority	Stabilizes the supply chain for chronic meds, reducing financial risk.

### Operational Pillar II: Digital health, supply chain, and data

#### Harnessing digital health for coverage expansion

Digital health, particularly mobile health (mHealth), leverages Somalia’s telecommunications network to overcome the barriers of distance, cost, and mobility. Digital health solutions can significantly increase care delivery for NCDs.

- **Telemedicine**: Services such as Hello! Caafi provides remote medical consultations, which are vital for chronic NCD/MH management when physical access is compromised.
- **Regulatory tools**: Digital platforms enforce standardization by integrating clinical guidelines directly into technology, bypassing systemic governance weaknesses.

#### Strengthening the chronic care supply chain

For life-long NCD patients, supply chain interruptions are often fatal. Resilience demands the continuous availability of essential NCD/MH medications. The EPHS-ISP mandates the standardization of essential drugs and a transition to dynamic forecasting linked to digital patient data.

#### Data and monitoring for accountability and resilience

- **Integrated HIMS**: The National Health Information Management System (HIMS) must be standardized, building on the use of the DHIS2 platform. Systematic data collection must be incorporated to assess NCD/MH prevalence and management outcomes.
- **Quality Metrics**: The EPHS-ISP mandates strengthening monitoring mechanisms, including balanced score cards (BSCs), standard monitoring checklists (SMCs), and the Health Facility Performance and Quality Assessment Tool (PQAT).

**Table 6:** Essential EPHS monitoring and accountability metrics| Location: Somalia. Table 6 lists various monitoring tools and mechanisms used to assess the effectiveness and quality of health services delivered under the EPHS, emphasizing their purpose at different health system levels

Tool/Mechanism	Level	Purpose in EPHS-ISP
Balanced Score Card (BSC)	National/State/District	Standardize monitoring of results; benchmark performance trends over time
Standard Monitoring Checklist (SMC)	Facility/District	Facilitate quarterly monitoring visits of EPHS health facilities
Performance/Quality Assessment Tool (PQAT)	Facility/DHMT	Assess and monitor implementation of health facility standards; develop quality improvement plans
Resource Mapping & Expenditure Tracking (RMET)	National/State	Identify financial gaps and improve expenditure efficiency annually
National Health Accounts (NHA)	National	Framework for collection and analysis of health expenditures (origins and utilization)

### Policy Pillar III: Sustainable financing and governance

#### Strategic health financing

To counter high out-of-pocket (OOP) spending (46%), Somalia needs to create significant fiscal space for health [10, 18]:

- **Increased Domestic Commitment**: Policy must target a measurable increase in government budget allocation for health (e.g., from 6% to 12% by 2030 in Somaliland) [20, 25].
- **Strategic Purchasing**: The Ministry of Health (MoH) will adopt a strategic purchasing approach incorporating a purchaser–provider split model, acting as the steward while contracting NGOs and the private sector as providers [8, 21]. A proportion of future fiscal space expansion should be directed toward strengthening health workforce remuneration and retention. International experience demonstrates that primary healthcare reforms achieve greater sustainability when workforce financing includes timely salary payments, rural service incentives, occupational protection measures, and continuous professional development opportunities. Within the Somalia EPHS framework, workforce financing should therefore be considered a strategic investment that complements service delivery expansion and improves long-term system performance.

#### Governance and implementation strategy

- **Geographic Harmonization**: Only a single NGO (service provider) is allocated per region to reduce transaction costs and improve accountability [8].
- **Delivery System Harmonization**: Vertically implemented interventions (TB, HIV, malaria, and nutrition) will be gradually integrated into the EPHS platform [21].
- **Flexible Service Delivery**: Flexible contracts are awarded to partners to develop creative solutions for service provision to hard-to-reach populations [8, 18].

### Economic findings: total costs and structural cost transformation (2020–2030)

**Fig. 2:**
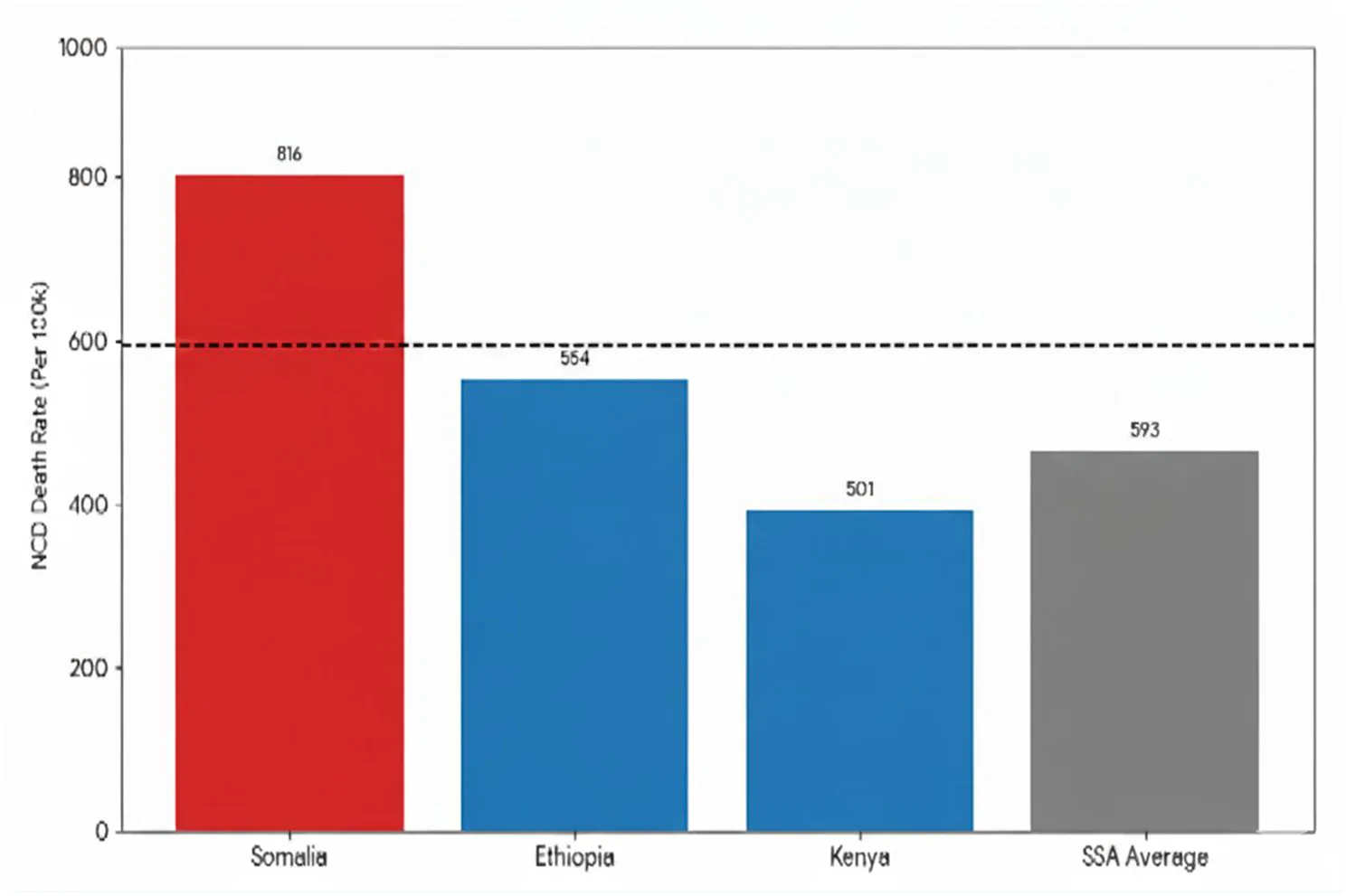
Regional NCD mortality benchmarking and investment rationale. (2024/2025). Source: Compiled from WHO Global Health Estimates (GHE) 2024 and National Health Data Centers*.*| Location: Somalia

The OHT projects the total investment required for the integrated EPHS to achieve 80% coverage by 2030 at $3.1 billion [13, 21]. This Fig. 2 provides the epidemiological context for the US$ 3.1 billion investment, illustrating the gap between current health expenditure and the projected requirements to mitigate the 80% of YLDs caused by noncommunicable diseases and mental health disorders [10, 15]. Somalia’s NCD mortality rate (816 per 100,000) according to WHO Global Health Estimates 2024 [16], supporting the need for substantial scale-up in chronic care expenditure.” significantly exceeds the SSA average (593 per 100,000), supporting the need for substantial scale-up in chronic care expenditure [16, 18]. Analysis of this investment across the five cost components reveals a highly concentrated cost structure, driven primarily by the new demands of chronic care.

#### Financial investment for EPHS scale-up: total costs and allocation

The $3.1 billion represents the total strategic capital required over the 11-year period. A more critical financial finding is the annual cost in the target year (2030), which is projected at $505 million. This establishes the perpetual recurrent financial commitment required to maintain a functioning 80%-coverage system. The breakdown of this total investment by the OHT cost component highlights a crucial structural shift, as detailed in Table 7.

The comparatively high proportion allocated to Medicines and Supplies reflects the chronic-care orientation of the EPHS integration strategy and should be interpreted within the context of Somalia’s fragile health system structure. Unlike conventional hospital-centered systems where workforce salaries dominate expenditure profiles, the Somalia EPHS model is built around decentralized PHC delivery, task-sharing approaches, and community-based service expansion through non-specialist cadres. Under this approach, human resource costs grow more gradually, whereas recurrent expenditures for chronic disease medications, insulin, antihypertensives, asthma therapies, psychotropic medicines, laboratory commodities, and long-term diagnostic supplies increase substantially as service coverage expands toward the 80% target. The OHT therefore projects a progressive shift from workforce-intensive acute care toward commodity-intensive chronic disease management. This financing pattern is consistent with the long-term operational demands of integrated NCD and mental health care in fragile and conflict-affected settings.

Unlike conventional hospital-centered health systems where human resources represent the dominant expenditure category, the Somalia EPHS chronic care expansion model produced a comparatively larger proportional allocation toward medicines and diagnostic supplies. This reflects several contextual assumptions within the OHT framework: (1) the reliance on task-sharing and community health workers reduced specialist salary expenditures; (2) long-term procurement requirements for antihypertensives, insulin, psychotropic medicines, diagnostics, and consumable supplies accumulated substantially over the projection period; and (3) major infrastructure expansion costs were not comprehensively modeled within the recurrent expenditure envelope. Consequently, medicine and supply costs emerged as the principal recurrent expenditure driver within the chronic disease scale-up scenario. Although medicines and supplies constitute the largest projected expenditure category, the allocation of approximately US$930 million to human resources remains a critical pillar of health system strengthening. Beyond workforce expansion, these investments provide an opportunity to improve retention, reduce attrition, support deployment to underserved areas, and strengthen motivation among nurses, midwives, community health workers, and other frontline providers responsible for delivering integrated NCD and mental health services.

**Table 7:** One Health Tool (OHT) projected total EPHS investment by cost component (2020–2030, US$)

OHT Cost Component	Total Projected Cost (2020–2030, US$ Millions)	Percentage of Total Investment	Annual Cost in 2030 (US$ Millions)	Policy Implications
Human Resources (HR)	930	30.0%	155	Task-sharing model; FHW recruitment and retention.
Medicines and Supplies	1,860	60.0%	290	Strategic purchasing, pooled procurement, quality assurance.
Infrastructure/Equipment	186	6.0%	30	PHC unit refurbishment, basic NCD diagnostic equipment.
Logistics and Operations	93	3.0%	15	Supply chain resilience, last-mile delivery mechanisms.
Programme Management	31	1.0%	5	M&E of coverage targets and financial tracking.
**Total**	**3**,**100**	**100.0%**	**505**	**Total investment required to achieve 80% coverage by 2030.**

*Source: EPHS 2020 OHT Costing Scenario (2020–2030) | Location: Somalia

Note: The relatively high proportion allocated to Medicines and Supplies reflects the chronic-care and commodity-intensive nature of integrated NCD and mental health service delivery under the EPHS scale-up scenario, including long-term pharmaceutical procurement, diagnostics, and recurrent supply requirements

#### Total projected costs and per capita expenditure

The OHT model projects a required total investment of US$3.1 billion from 2020–2030, requiring annual per capita spending to increase from $7.4 in 2020 to $33.3 by 2030. The projections for per capita costs and total investment from 2020–2030 are detailed in Table 8. This table outlines the expected increase in funding needs, emphasizing the importance of strategic health financing to achieve the UHC target.”

**Table 8:** EPHS 2020 per capita costs and total projected costs (US$ million)

Year	Per Capita Cost (US$)	Grand Total Cost (US$ million)
2020	7.4	104.54
2025	14.1	231.56
2030	33.3	625.47
**Total (2020–2030)**	**N/A**	**3106.03**

*Source: EPHS 2020 OHT Costing Scenario (2020–2030). Location: Somalia

#### Composition of costs: definitive shift to chronic care supplies

The most significant finding is the structural shift in cost composition: the cost of medicines and supplies is projected to increase from 34% of the total in 2020 to 60% by 2030 [10, 13]. This confirms that the system will be dominated by the sustained, consumption-based requirements of managing chronic NCD and MH patients. Table 9 outlines the projected costs for human resources, infrastructure, medicines, logistics, and program costs, illustrating the shift toward chronic care management in Somalia’s health system.

**Table 9:** Composition of EPHS 2020 implementation costs (US$ million) from 2020–2030

Cost Component	2020 (US$ Million)	2030 (US$ Million)	Total (2020–2030) (US$ Million)
Total Human Resources (Salaries)	48.22	118.58	895.34
Total Infrastructure Cost	10.49	13.04	128.93
**Total Medicines and Supplies Cost**	**36.03**	**375.06**	**1594.26**
Total Logistics Cost	9.63	117.33	474.22
Total Programme Costs	0.18	1.46	13.29
**Grand Total**	**104.54**	**625.47**	**3106.03**

*Source: EPHS 2020 OHT Costing Scenario (2020–2030) | Location: Somalia

**Table 10:** Percentage distribution of EPHS cost components (visualizing the shift). Table 10 visualizes the changing distribution of costs among various components of the Essential Package of Health Services, highlighting the increasing importance of medicines and supplies over time

Cost Component	2020 (%)	2030 (%)	Change (%)	Implication for Chronic Care
Human Resources (Salaries)	46%	19%	-27%	Decreasing relative importance post initial investment.
**Medicines and Supplies**	**34%**	**60%**	**+ 26%**	**Dominant driver by 2030; reflects sustained chronic medication needs.**
Infrastructure	10%	2%	-8%	Decreasing relative importance post initial rehabilitation.
Logistics	9%	19%	+ 10%	Increase proportional to drug/supply distribution scale.

*Source: EPHS 2020 OHT costing scenario (2020–2030) | Location: Somalia

#### Dynamic financial analysis: structural cost shift

**Fig. 3:**
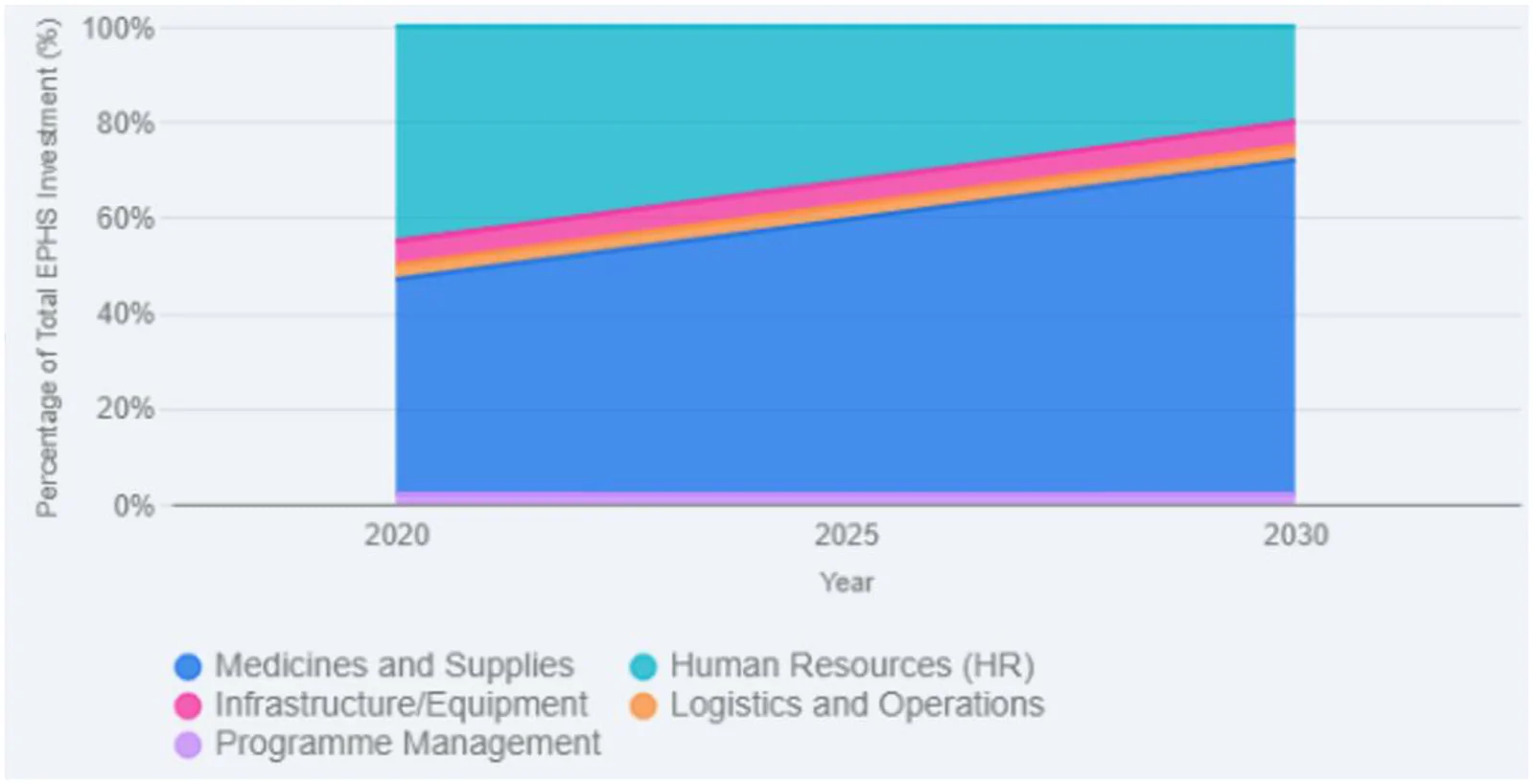
OHT projected structural cost composition shift: Increasing share of chronic care medicines and supplies (2020–2030). (Stacked Area Chart) (Projected proportional distribution of EPHS cost components, Somalia, 2020–2030.”)

Figure 3 visualizes this transition, illustrating the fundamental and permanent alteration of the EPHS financing model. In the early years, costs were balanced. By the middle of the decade, the HR band had contracted (relatively) as the task-sharing model saturated, whereas the Medicines and Supplies band had begun steep, continuous expansion [11, 13]. By 2030, Medicines and Supplies dramatically dominate the cost composition, demonstrating the transition to a pharmaceutical logistics and financing platform.

#### Resource allocation, efficiency, and dominant cost drivers

The decentralized nature of the EPHS is reflected in the allocation, with health centers (HCs) consuming the largest absolute resource amount (Table 11), confirming their status as the PHC delivery hub.

**Table 11:** Total EPHS implementation cost by facility type (US$ million) - selective years

Facility Type	2020 Cost (US$ Million)	2025 Cost (US$ Million)	2030 Cost (US$ Million)
Community	3.8	13.5	28.6
Primary Health Unit (PHU)	8.4	18.9	57.3
**Health Centre (HC)**	**39.1**	**89.9**	**259.0**
District Hospital (DH)	27.9	59.0	144.9
Regional Hospital/Office	23.5	47.0	107.2

Source: EPHS 2020 OHT Costing Scenario (2020–2030) | Location: Somalia

Efficiency gains from the task-sharing model are confirmed by the projected decline in the average cost per patient visit (CPPV) across most facility types by 2030 (Table 12), signifying successful diversion of routine care to lower-cost settings.

**Table 12:** EPHS implementation cost per patient visit by facility type (US$)

Facility Type	2020 Cost Per Patient Visit (US$)	2030 Cost Per Patient Visit (US$)	Average Cost Per Patient Visit (2020–2030) (US$)
Community	0.45	0.32	0.40
Primary Health Unit (PHU)	0.42	0.44	0.37
**Health Centre (HC)**	**3.41**	**2.51**	**2.63**
**District Hospital (DH)**	**4.25**	**2.42**	**2.81**
Regional Hospital (RH)	14.39	9.37	10.78

Source: EPHS 2020 OHT Costing Scenario (2020–2030) | Location: Somalia

#### NCD and nutrition cost driver analysis

By 2030, the financial dominance of chronic NCD medication was clearly established. At baseline (Table 13), costs were driven by acute nutrition. By 2030 (Table 14), NCD management interventions (asthma and diabetes) will become the undisputed top two cost drivers, consuming 21% of the total drug/supply budget.

**Table 13:** Top 5 drug and supply cost drivers at baseline (2020)

Intervention	Cost (US$)	Cost % of Total Drugs/Supplies
Supplementary feeding for pregnant women with MAM	3,214,454	9%
Management of asthma	2,928,811	8%
Management of diabetes	2,592,647	7%
Supplementary feeding for lactating women with MAM	2,539,221	7%
Maternal nutrition assessment, counseling, and MMN	1,420,753	4%

*Source: EPHS 2020 OHT Costing Scenario (2020–2030) | Location: Somalia

**Table 14:** Top 5 drug and supply cost drivers at the end line (2030)

Intervention	Cost (US$)	Cost % of Total Drugs/Supplies
Management of diabetes	27,859,289	10%
Management of asthma	31,437,983	11%
Management of moderate acute malnutrition (children)	12,829,574	5%
Abdominal pain, gastritis, and GI bleeding	10,613,015	4%
Management of injuries	9,895,010	4%

Source: EPHS 2020 OHT Costing Scenario (2020–2030) | Location: Somalia

### Projected health impact and return on investment

**Fig. 4:**
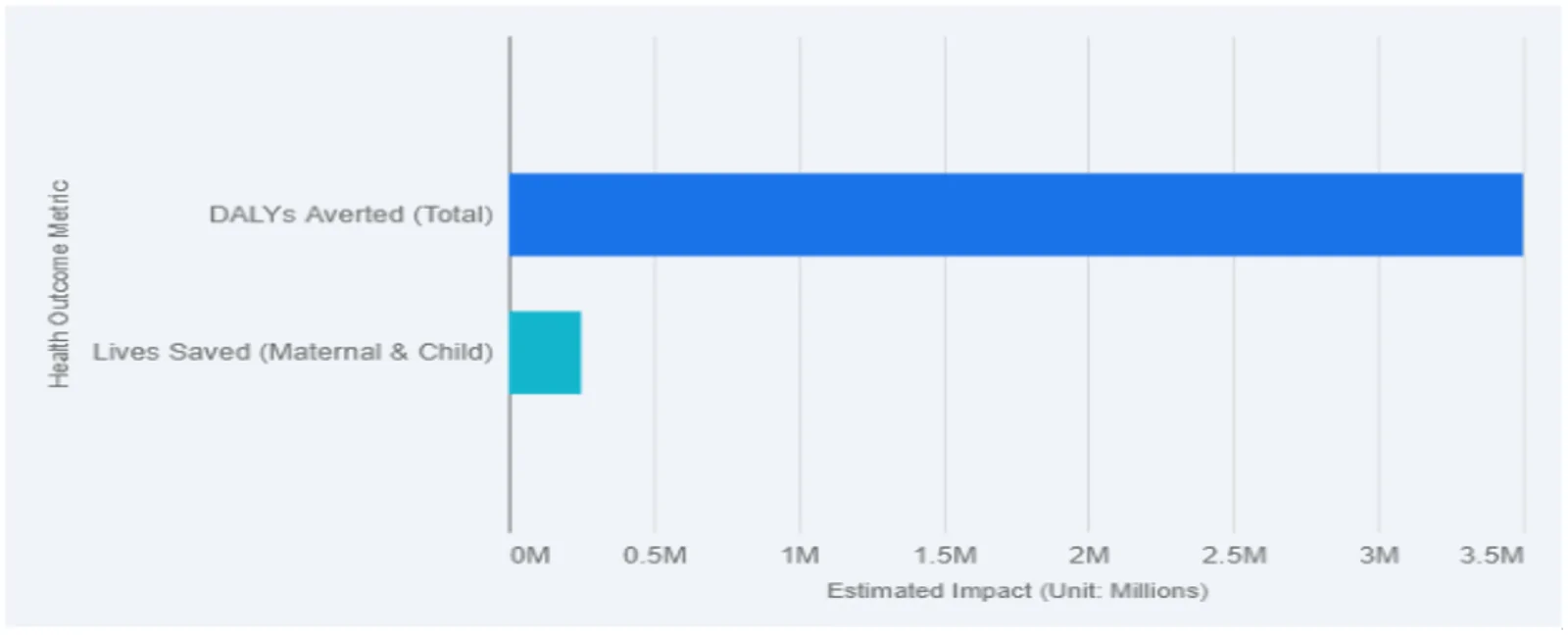
Projected EPHS impact: Estimated maternal and child lives saved and DALYs averted due to integrated care scale-up, Somalia, 2030. (Bar Chart) (Projected proportional distribution of EPHS cost components, Somalia, 2020–2030.”

The final set of results quantifies the public health return expected from the strategic $3.1 billion investment, suggesting the potential effectiveness of the integrated model” across both mortality (YLL) and morbidity (YLD) metrics as shown Fig. 4.

#### Quantifying health gains

- **Estimated Maternal and Child Lives Saved**: The significant number of lives saved (e.g., 250,000) confirms that the EPHS scale-up succeeds in its fundamental goal of addressing high CMNN-related mortality, which historically represents up to 68.8% of total disability-adjusted life years and dominant country YLLs [15, 26].
- **Total DALYs Averted**: The massive volume of DALYs Averted (e.g., 3.5 million) represents the true success of the integration strategy. Since this metric captures both YLL and YLD, the sheer magnitude of the averted DALYs demonstrates the substantial value generated by reducing the chronic disability burden (80% of total YLDs) through effective NCD/MH management.

#### Projected impact on mortality and morbidity

The scale-up of EPHS 2020 interventions provides a comprehensive method to tackle multi-sectoral health deficiencies within the population [8, 18], suggesting substantial potential return on the US$3.1 billion investment. The projected reductions in mortality rates are summarized in Table 15, which outlines the anticipated decreases in maternal and child mortality as a result of the implementation of the EPHS 2020.

**Table 15:** Projected reduction in child and maternal mortality rates (2020 vs. 2030)

Indicator (per 1,000 or 100,000 LBs)	Baseline (2020)	Projected (2030)	Expected Reduction (%)
Maternal Mortality Ratio (MMR) (per 100,000 LBs)	692	331.6	~ 52%
Neonatal Mortality Rate (per 1,000 LBs)	38	20	~ 47%
Under-Five Mortality Rate (U5MR) (per 1,000 LBs)	122	63	~ 48%

Source: EPHS 2020 OHT Impact Projections | Location: Somalia

#### Nutritional and chronic disease outcomes

Significant investment in nutrition and NCD interventions is expected to dramatically improve population health metrics. The NCD interventions are projected to increase the number of healthy life years lived and reduce the annual probability of death due to NCDs in the 30–70 year age bracket. Projected reductions in malnutrition are detailed in Table 16.

**Table 16:** Projected reduction in global stunting and wasting rates (2020 vs. 2030)

Indicator	Baseline (2020)	Projected (2030)	Expected Reduction (Percentage Points)
Global Stunting Rate (Children 0–59 months)	41.0%	36.6%	4.4%
Global Wasting Rate (Children 0–59 months)	13.5%	6.3%	7.2%

Source: EPHS 2020 OHT Impact Projections **| **Location: Somalia

## Discussion

### Epidemiological mandate as a justification for systemic transformation

The results from the GBD 2019 analysis (Tables 3 and 4) present an undeniable case for the strategic pivot institutionalized in the EPHS 2020 [5, 15]. The Somali health system has historically, and justifiably, focused on acute mortality (YLLs), which are dominated by CMNN diseases [15]. However, the data clearly show that this focus has left the population’s primary source of suffering—chronic disability (YLD)—almost entirely unaddressed [15]. While uncertainty remains regarding the precise magnitude of mental health prevalence estimates in Somalia, the overall epidemiological pattern consistently demonstrates that chronic disability is increasingly driven by noncommunicable and mental health conditions rather than acute infectious mortality alone [15, 16]. The evidence therefore supports a scenario-based health systems interpretation: communicable, maternal, neonatal, and nutritional disorders remain dominant drivers of premature mortality (YLLs), whereas mental health and other chronic conditions disproportionately contribute to prolonged disability (YLDs), reduced productivity, and long-term functional impairment [15]. Within this framework, the integration of WHO-PEN and mhGAP into primary health care is justified not because mental health conditions constitute the majority of the total disease burden, but because they represent a substantial and historically neglected component of chronic disability requiring continuous care delivery within fragile settings [1, 2, 7].

### Medicines as infrastructure: the new financial paradigm

The most profound finding of this analysis is the structural cost transformation observed in the OHT projections (Table 10; Fig. 3), where Medicines and Supplies increase from 34% of total EPHS expenditure in 2020 to approximately 60% by 2030, while the relative proportion allocated to Human Resources declines over time [13, 14]. Although this distribution may initially appear atypical compared with conventional health systems—where salaries commonly represent the dominant expenditure category—it reflects the unique epidemiological, operational, and service-delivery realities of Somalia’s fragile context and the methodological assumptions embedded within the OHT chronic care expansion scenario [10, 13].

First, the EPHS 2020 model relies heavily on task-sharing and decentralized delivery through nurses, midwives, and Marwo Caafimaad Female Health Workers (FHWs), substantially reducing dependence on expensive specialist-led care models [7, 11, 21]. Second, the scale-up strategy prioritizes long-term management of chronic diseases such as diabetes, asthma, hypertension, and mental health disorders, all of which require continuous pharmaceutical treatment, diagnostics, and recurrent commodity procurement over the life course of patients [7, 17, 25]. Third, Somalia’s baseline workforce density remains extremely low, limiting the immediate absorptive capacity for large-scale salary expansion despite increasing service coverage targets [3].

Consequently, the OHT projections generate a financing structure in which recurrent pharmaceutical and diagnostic supply costs expand more rapidly than workforce costs during progressive scale-up [13]. Similar expenditure transitions have been reported in several low- and middle-income countries undergoing epidemiological transition toward chronic disease management, particularly where PHC-based NCD integration and task-shifting strategies are emphasized [11, 12]. Therefore, the 60% allocation to medicines and supplies should not be interpreted as a conventional salary-to-workforce financing model, but rather as the projected financial signature of a chronic-care-oriented PHC system operating within a fragile and supply-constrained environment [10, 13]. This finding additionally highlights that the principal long-term vulnerability of Somalia’s future PHC system may shift from workforce availability toward sustainable pharmaceutical financing, procurement efficiency, and supply-chain continuity [17, 18].

### Human Software” solution and task-shifting for resilience

With respect to resilience via task-shifting and digital health, the $3.1 billion plan is only financially viable because of its operational strategy [13, 21]. The model wisely avoids a specialist-led, high-cost HR approach, which is impossible in a fragile context and instead invests in " heavily in human resource training and optimization” [21]. The task shifting of complex NCD/MH protocols (WHO-PEN/MhGAP) to nonspecialist nurses and, crucially, to the “Marwo Caafimaad” FHWs is the engine of the EPHS [7, 11]. This approach involves HR costs (Table 7) and is the only feasible way to decentralize knowledge and close the coverage gap [11].

This localized operational framework “human software” is managed and scaled via digital tools [18]. The use of mHealth platforms for remote consultation and HIMS/DHIS2 for data management provides the necessary quality control and monitoring that the weak regulatory environment [1]. This combination of task-shifting and digital oversight is the core of the resilience model, allowing the system to bypass physical access barriers and systemic governance weaknesses [4, 12]. However, task-sharing should not be interpreted as a substitute for adequate workforce investment. Global evidence demonstrates that task-sharing programmes achieve sustainable results only when accompanied by fair compensation, supportive supervision, manageable workloads, and opportunities for professional advancement. In Somalia, expanding responsibilities for nurses, midwives, and Marwo Caafimaad Female Health Workers without corresponding improvements in remuneration and working conditions may undermine motivation and contribute to workforce attrition. Consequently, future EPHS financing reforms should incorporate retention-focused policies including performance-based incentives, hardship allowances for underserved areas, occupational safety protections, and career development pathways. Such investments are critical for translating workforce expansion into long-term health system resilience and improved service quality.

### Strategic integration: the “Dual Mandate” impact: justifying investment (as an Investment Driver)

The projected health impact (Section “Projected health impact and return on investment”) reveals the strategic value of the EPHS 2020 [7, 21]. The analysis presents a long-term strategic investment scenario intended to guide progressive health system strengthening and resource mobilization for integrated NCD and mental health services [13]. By demonstrating enormous, quantifiable, and highly fundable gains in traditional MCH outcomes, it satisfies a dual requirement [26]. These MCH gains (which address YLLs) effectively serve as the Strategic Integration Framework (a “Trojan horse”) to build the platform and secure financing for NCD/MH interventions (which address YLDs) [15, 19]. The massive number of DALYs averted (Fig. 3) confirms that the strategy successfully addresses both mandates, validating chronic care investment as a driver of human capital accumulation and national productivity [2, 15].

### Inherent fragility and key vulnerability (Supply Chain)

While the OHT projections provide an important strategic framework for long-term health sector planning, the scale and pace of the modeled expansion must be interpreted cautiously within Somalia’s operational realities [13, 14]. Increasing service coverage from the current UHC-SCI baseline toward universal targets within a short implementation window may not be fully achievable under existing fiscal and institutional constraints [2]. Somalia continues to face major barriers including low per-capita health expenditure, heavy donor dependency, insecurity, workforce shortages, supply chain fragility, and limited absorptive capacity [3, 10, 18, 25].

Therefore, a phased implementation strategy prioritizing high-burden and high-impact interventions may represent a more feasible pathway toward progressive UHC expansion [2, 18]. The relatively limited volume of nationally representative implementation research further reflects the structural challenges of conducting large-scale health systems research in settings affected by prolonged conflict, institutional fragility, and fragmented health information systems [1, 4]. Despite the robust strategy, the discussion must acknowledge critical vulnerabilities [19]. The EPHS 2009 failed because of fragmentation and implementation and selective programatic by partners [8]. The success of EPHS 2020 depends entirely on the MoH’s ability to enforce the new governance model—specifically, the “purchaser–provider split” and “geographic harmonization”—to force the delivery of the entire package, not just the easily funded vertical programs [21].

An additional vulnerability concerns workforce sustainability. While the OHT projections allocate approximately US$930 million to human resources, workforce financing should not focus exclusively on recruitment and deployment. Persistent challenges related to inadequate remuneration, delayed salary payments, difficult working environments, insecurity, and migration of skilled personnel remain significant threats to implementation success. Without complementary investments in compensation, workforce welfare, occupational safety, and professional development, the projected gains from task-sharing and decentralized service delivery may not be fully realized. Strengthening workforce retention should therefore be regarded as a central component of resilience building rather than a secondary administrative concern. Furthermore, the OHT model allocates only 3.0% ($93 M) of the total budget to logistics and operations (Table 7) [13]. This represents a critical structural bottleneck for a system that is 60% dependent on its pharmaceutical supply chain, operating in an environment marked by persistent insecurity [10, 17, 19]. This budget line represents a significant risk, as any failure (“a major operational vulnerability”) because any disruption in the pharmaceutical supply chain could substantially reduce the effectiveness of the projected medication investment [17].

### Strengths and limitations

This study has several strengths. First, it integrates health systems analysis with prospective economic modelling to examine the implications of incorporating noncommunicable disease (NCD) and mental health services into Somalia’s primary health care (PHC) system. This approach enabled the simultaneous assessment of disease burden, service delivery requirements, workforce needs, and financing implications within a single analytical framework.

Second, the study synthesizes evidence from multiple sources, including national policy documents, administrative reports, health financing data, and epidemiological estimates. The use of diverse data sources enhanced the contextual relevance of the analysis and provided a comprehensive assessment of health system priorities in a fragile and conflict-affected setting. Furthermore, application of the OneHealth Tool (OHT) enabled a systematic estimation of resource requirements associated with implementing the Essential Package of Health Services (EPHS 2020), including the integration of WHO-PEN and mhGAP interventions within PHC.

Third, the costing analysis was informed by country-specific parameters derived from Somalia’s National Health Accounts and other national planning documents. This strengthens the policy relevance of the findings by grounding projections in the national health financing and service delivery context.

Several limitations should be considered. First, the OHT employs a deterministic modelling framework that relies on assumptions regarding service coverage expansion, implementation capacity, and resource availability. Consequently, the projections should be interpreted as strategic planning estimates rather than forecasts of future expenditure or health outcomes.

Second, limitations in the availability of nationally representative data required the use of secondary epidemiological estimates, including data from the Global Burden of Disease study. Although these sources provide the best available evidence for planning purposes, they may not fully capture subnational variation or the health needs of displaced, nomadic, and other underserved populations.

Third, the model does not fully account for contextual uncertainties that may affect implementation; including insecurity, political instability, climate-related shocks, inflation, supply-chain disruptions, and changes in external financing. In addition, although workforce costs were included, the analysis did not explicitly model workforce dynamics such as retention, migration, productivity, or responses to compensation and incentive reforms.

Despite these limitations, the study provides an evidence-informed assessment of the resource requirements and health system implications of integrating NCD and mental health services within Somalia’s PHC platform and offers a useful basis for future planning, investment, and research.

## Conclusion, policy recommendations and phased roadmap

### Conclusion

This study highlights the growing importance of noncommunicable diseases (NCDs) and mental health conditions within Somalia’s evolving disease burden and underscores the need for health system responses that extend beyond traditional priorities focused on acute and communicable diseases. The findings suggest that integrating NCD and mental health services into the primary health care (PHC) platform represents a practical and strategically important approach to strengthening health system resilience and advancing progress toward Universal Health Coverage (UHC).

The Essential Package of Health Services (EPHS 2020) provides a coherent policy framework for this transition by promoting more integrated, equitable, and people-centered service delivery. The OneHealth Tool (OHT) projections indicate that substantial investment will be required to expand coverage and strengthen service readiness over time. While the estimated resource requirements should be interpreted as long-term planning scenarios rather than fixed expenditure commitments, they offer a useful evidence base for prioritizing investments in service delivery, medicines, diagnostics, health infrastructure, and workforce development.

The study also emphasizes that successful implementation will depend on factors extending beyond financing alone. Sustained improvements in health workforce capacity, retention, supervision, supply chain performance, governance, and institutional coordination will be critical to translating policy ambitions into effective service delivery. Given Somalia’s fragile operating environment, a phased and adaptive implementation approach, aligned with available resources and system capacity, is likely to be both necessary and appropriate.

Overall, the findings support the view that strengthening PHC through the integration of NCD and mental health services can contribute to a more responsive and resilient health system capable of addressing current and emerging population health needs. Although important implementation challenges remain, EPHS 2020 provides a valuable roadmap for progressively expanding access to essential services, improving health outcomes, and advancing Somalia’s long-term UHC objectives. The experience and lessons generated through this process may also offer relevant insights for other fragile and conflict-affected settings seeking to strengthen PHC and address the rising burden of chronic disease.

### Policy recommendations

#### Establishing a Centralized Pooled Procurement Mechanism

Given that “Medicines and Supplies” account for 60% of all costs ($1.86B), this is the single most critical recommendation. The MoH must immediately establish a national, transparent mechanism for strategic purchasing and pooled procurement. This is essential to achieve economies of scale, ensure drug quality, and manage the $290 million annual recurrent pharmaceutical bill projected for 2030.

#### Professionalize and Fund the FHW Cadre

The task-shifting model is the core “human software” of the EPHS. This cannot be a volunteer-based or ad hoc program. The MoH and its partners must create formal, funded pathways for the recruitment, training, supervision, and retention of the “Marwo Caafimaad” FHWs to ensure the stability and quality of the entire community-level service delivery model.

#### Mandate and Enforce Governance Harmonization

To prevent the failure of the EPHS 2009, the MoH must rigorously enforce the “purchaser–provider split” and the “geographic harmonization” (single-NGO-per-region) model. This governance mechanism is the only tool available to force the integration of NCD/MH care and prevent partners from fragmenting the system.

#### Derisk the Chronic Care Supply Chain

The 3.0% budget for logistics is a key vulnerability. A portion of new funding must be earmarked to build a resilient (secure, redundant, and digitally tracked) supply chain. The HIMS/DHIS2 platform must be fully integrated with this supply chain for “dynamic forecasting” to ensure that the $1.86B in medicines reaches the last mile without interruption.

#### Securing Sustainable and Domestic Financing

While donor funding is essential, the $505 million annual recurrent cost (by 2030) is not sustainable without domestic commitment. The MoH must leverage the NHA and RMET tools **(**Table 6**)** to advocate for phased increases in domestic budget allocation for health, in line with the Somaliland target (6% to 12%).

#### Establish a National Health Workforce Retention and Incentive Framework

The Federal Ministry of Health and Human Services, together with Federal Member States and development partners, should establish a comprehensive National Health Workforce Retention and Incentive Framework aimed at strengthening recruitment, retention, motivation, and performance across all levels of the health system. Priority interventions should include equitable remuneration, timely salary payments, rural hardship allowances, occupational safety protections, supportive supervision, continuing professional development, and structured career advancement opportunities.

### Phased implementation roadmap, the OHT model’s projections imply a three-phased implementation roadmap

**Phase 1: Build-Up (2020–2024)**: The primary focus is on inputs. This phase includes finalizing governance contracts (geographic harmonization), training the workforce (FHWs, nurses) in the WHO-PEN/MhGAP protocols, and refurbishing PHC units with essential NCD/MH diagnostic tools.

**Phase 2: Transition/Convergence (2025–2027)**: The focus shifts to scale-up and financial transition. As service coverage expands, financial risk shifts decisively to the pharmaceutical supply chain. The Pooled Procurement Fund must be fully operational, and mHealth/HIMS data systems **(**Table 6**)** must be scaled to manage the growing patient load and commodity flow.

**Phase 3: Maintenance/Dominance (2028–2030)**: The focus is on sustainability and quality. The system approaches the 80% coverage target. The primary operational function becomes the perpetual management of the $290 million annual recurrent drug bill. Success is measured by the system’s ability to maintain continuity of care and monitor quality via the BSC and PQAT tools (Table 6).

## Data Availability

The datasets supporting the conclusions of this article are derived from publicly available national strategic documents and global health estimates cited throughout the manuscript, including the EPHS 2020 Costing Scenario (One Health Tool), Global Burden of Disease estimates 2019, and Federal Ministry of Health policy documents. These public sources are fully referenced in the bibliography.

## Abbreviations

BSC: Balanced scorecard
CMNN: Communicable, maternal, neonatal, and nutritional
DALY: Disability-Adjusted Life Years
DHIS2: District Health Information Software 2
EPHS: Essential Package of Health Services
FHW: Female Health Workers
FMOH: Federal Ministry of Health
GBD: Global Burden of Disease
HIMS: Health Information Management System
MhGAP: WHO Mental Health Gap Action Programme
MH: Mental Health
MOH: Ministry of Health
NCD: Noncommunicable Diseases
NHA: National Health Accounts
OHT: One Health Tool
OOP: Out-of-Pocket Payments
PHC: Primary Health Care
PQAT: Performance/Quality Assessment Tool
PRISMA: Preferred Reporting Items for Systematic Reviews and Meta-Analyses
RMNCAH: Reproductive, Maternal, Newborn, Child, and Adolescent Health
SMC: Standard Monitoring Checklist
UHC: Universal Health Coverage
WHO: World Health Organization
WHO-PEN: WHO Package of Essential Noncommunicable Disease Interventions
YLD: Years Lived with Disability
YLL: Years of Life Lost
